# Association analysis of photoperiodic flowering time genes in west and central African sorghum [*Sorghum bicolor *(L.) Moench]

**DOI:** 10.1186/1471-2229-12-32

**Published:** 2012-03-07

**Authors:** Sankalp U Bhosale, Benjamin Stich, H Frederick W Rattunde, Eva Weltzien, Bettina IG Haussmann, C Thomas Hash, Punna Ramu, Hugo E Cuevas, Andrew H Paterson, Albrecht E Melchinger, Heiko K Parzies

**Affiliations:** 1 Institute of Plant Breeding, Seed Science, and Population Genetics, University of Hohenheim, 70593 Stuttgart, Germany; 2 Max Planck Institute for Plant Breeding Research, 50829 Köln, Germany; 3 International Crops Research Institute for the Semi-Arid Tropics (ICRISAT) - Bamako, BP 320 Bamako, Mali; 4 ICRISAT - Sadoré, BP 12404 Niamey, Niger; 5 ICRISAT - Patancheru, Hyderabad 502324, Andhra Pradesh, India; 6 Plant Genome Mapping Laboratory, University of Georgia, Athens GA 30602, USA; 7 U.S. Dept. of Agriculture, Agricultural Research Service, Tropical Agriculture Research Station, 2200 P.A. Campos Ave., Mayaguez P.R. 00680, Puerto Rico

## Abstract

**Background:**

Photoperiod-sensitive flowering is a key adaptive trait for sorghum (*Sorghum bicolor*) in West and Central Africa. In this study we performed an association analysis to investigate the effect of polymorphisms within the genes putatively related to variation in flowering time on photoperiod-sensitive flowering in sorghum. For this purpose a genetically characterized panel of 219 sorghum accessions from West and Central Africa was evaluated for their photoperiod response index (PRI) based on two sowing dates under field conditions.

**Results:**

Sorghum accessions used in our study were genotyped for single nucleotide polymorphisms (SNPs) in six genes putatively involved in the photoperiodic control of flowering time. Applying a mixed model approach and previously-determined population structure parameters to these candidate genes, we found significant associations between several SNPs with PRI for the genes *CRYPTOCHROME 1 *(*CRY1-b1*) and *GIGANTEA *(*GI*).

**Conclusions:**

The negative values of Tajima's D, found for the genes of our study, suggested that purifying selection has acted on genes involved in photoperiodic control of flowering time in sorghum. The SNP markers of our study that showed significant associations with PRI can be used to create functional markers to serve as important tools for marker-assisted selection of photoperiod-sensitive cultivars in sorghum.

## Background

Sorghum [*Sorghum bicolor *(L.) Moench] is a major staple crop and source of income for millions of people in Western and Central Africa (WCA). The success of sorghum production is determined to a considerable extent by the appropriateness of the flowering time for the specific production environment. The highly variable sowing dates, due in part to erratic onset of the rainy season, present an important challenge since grain maturity needs to occur at a more fixed calendar date to coincide with the end of the rainy period for successful grain filling and pest avoidance [1]. Thus, photoperiod-sensitive flowering responses of sorghum in WCA enhance adaptation by enabling more fixed maturity dates despite variable sowing dates [2-4].

The transition of plant growth from vegetative to generative stage is the primary determinant of flowering time in crops of determinant growth type such as sorghum. The degree to which varieties can adjust this onset of panicle initiation with differing sowing dates, and photoperiod conditions, is called photoperiodic flowering response [5]. Photoperiod sensitivity triggers panicle initiation in short-day (SD) plants such as sorghum when they sense an appropriate decrease in day length [6].

The molecular basis of flowering time has been extensively studied in *Arabidopsis thaliana *where mutant plants with an altered flowering phenotype were analyzed for their flowering behavior under laboratory conditions. As a result, four important pathways regulating floral induction have been identified: the photoperiod (long-day (LD) promotion) pathway, gibberellic-acid promotion pathway, vernalization pathway, and autonomous pathway [7-9]. A basic understanding of the molecular complexity of flowering time in important agronomic species with large genomes such as maize (*Zea mays *L.), wheat (*Triticum aestivum *L.), barley (*Hordeum vulgare *L.), and pearl millet (*Pennisetum glaucum *(L.) R. Br.) has been facilitated by comparative use of floral pathways from *A. thaliana *(for review see, [9-11]). Flowering time genes and sequences can be used by breeders for the development of molecular markers or for targeted genetic modification of flowering time.

### Current knowledge on genetics of photoperiod-sensitive flowering

**Figure 1:**
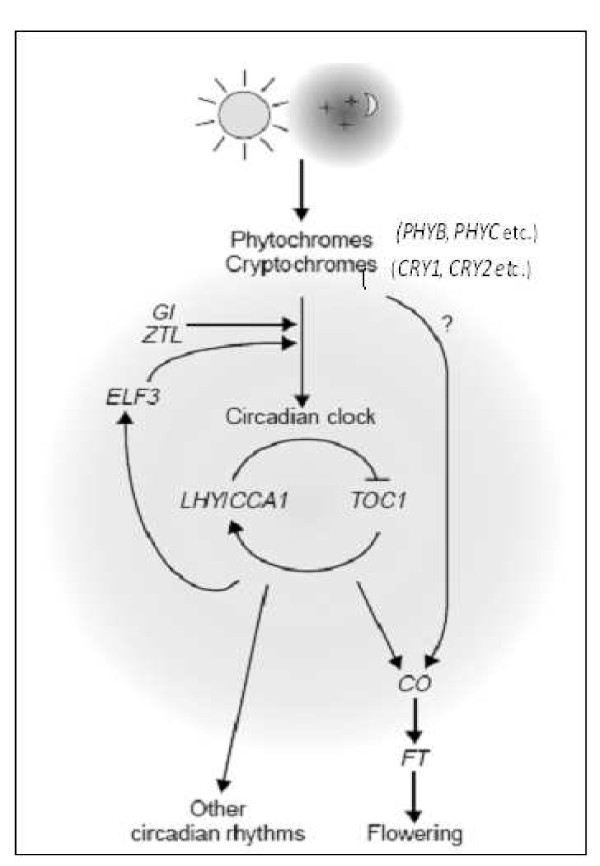
**A simplified model of flowering mediation by photoperiod in *Arabidopsis *(modified from Izawa et al**. [9]).

Since Bünning [12] first proposed that the photoperiodic time-keeping mechanism is associated with the circadian clock, there has been a considerable amount of research on the photoperiod pathway. The basis of day-length measurement is the interaction of an external light signal with the circadian rhythm [6]. In the photoperiod-sensitive flowering process (Figure 1), light signals are perceived by photoreceptors involved in the resetting of the circadian clock, with the result that plants respond to the light and dark cycles [13]. Genes such as *CIRCADIAN CLOCK ASSOCIATED1 *(*CCA1*), *LATE ELONGATED HYPOCOTYL *(*LHY*), and *TIMING OF CAB EXPRESSION1 *(*TOC1*) are the core components of the central oscillator of the circadian system. The oscillator determines the phase of *CONSTANS *(*CO*) transcription [14]. *CO *is an important gene that links the circadian clock to flowering [15], and it induces the transcription of *FLOWERING LOCUS T (FT) *to promote flowering [11,16]. Recent research has shown that the *FT *protein in *Arabidopsis *and corresponding proteins in other plants are an important part of the florigen [17,18], which is a leaf-generated mobile flowering signal initiating floral morphogenesis at the shoot apex [17,19].

### Status of research on flowering time genes in sorghum

A series of six maturity genes have been recognized to affect flowering time and photoperiodic flowering response in sorghum: *Ma_1_*, *Ma_2_*, *Ma_3_*, *Ma_4_*, *Ma_5_*, and *Ma_6 _*[20,21]. The first four maturity genes inhibit flowering under LD conditions but allow early flowering under short day conditions. Of these first four genes, *Ma_1 _*causes the greatest sensitivity to LD conditions. In contrast, *Ma_2_*, *Ma_3_*, and *Ma_4 _*generally have more modest effects on sensitivity to LD conditions [20]. Kouressy et al. [22] showed that photoperiod sensitivity was affected by dominant alleles of one major gene, equivalent to the *Ma_5 _*or *Ma_6 _*maturity loci identified by Aydin et al. [23]. Several other studies report on sorghum photoperiodic flowering [24-26]. These studies highlighted the role of phytochromes as an important gene family. Childs et al. [27] demonstrated that the *Ma_3 _*gene is synonymous to *PHYB *and sequenced other phytochromes such as *PHYA *and *PHYC*. It is interesting that mutations in sorghum *Ma_3 _*and *A. thaliana PHYB *both reduce sensitivity to non-inductive day-lengths [28,29]. Recently, positional cloning identified *Ma1 *as a putative pseudoresponse regulator protein 37 (PRR37), which acts as inhibitor of *CO *and floral activator genes [30]. Bhattramakki et al. [31] reported that primers for SSR marker *Xtxp320 *are derived from the *PHYB *sequence, but this sequence variation was not detected by White et al. [32] in studies of the *PHYB *sequence from several diverse sorghum accessions. There are indications that the *Xtxp320 *(*PHYB*) primer pair detects more than one locus as there are reports of *Xtxp320 *mapping to SBI-01 (where *PHYA*, *PHYB *and *PHYC *are located) and/or SBI-10 (e.g., [33]). However, to our knowledge, there has not been a study analyzing systematically the effect of candidate genes (CGs) from the photoperiod sensitivity pathway on photoperiodic flowering response in sorghum.

Advanced plant breeding techniques such as MAS have the potential to accelerate the selection process substantially [34,35]. Functional markers are the state-of-the-art molecular tools that minimize the risk of recombination between marker and QTL alleles [36]. Association studies based on linkage disequilibrium offer a new possibility to identify marker-trait associations (cf. [37]).

In this study we examined a panel of sorghum accessions from West Africa established expressly to represent the range of photoperiodic response. The objectives of our study were to (i) characterize the photoperiodic flowering response of these sorghum accessions under field conditions, and (ii) investigate the association between variation for photoperiodic sensitivity for flowering time and polymorphisms in six partially amplified genes putatively related to variation in flowering time in sorghum [*CRYPTOCHROME *1 (*CRY1*; Sb04g023680), *CRYPTOCHROME *2 (*CRY2*; Sb06g018510), *LATE ELONGATED HYPOCOTYL *(*LHY*; Sb04g031590), *GIGANTEA *(*GI*; Sb03g003650), *HEADING DATE *6 (*HD6; *Sb02g001110), and *Dwarf8 *(*SbD8*; Sb01g010660)].

## Results

### Phenotypic evaluation

Analysis of the field data on flowering time showed that sorghum accessions of our study exhibited a wide range of photoperiodic response. The days to 50% flowering (DFL50%) of sorghum accessions sown on 10 June ranged from 47 to 141, and those sown on 10 July from 44 to 117 days, respectively (Table 1). The PRI for the accessions ranged from -7 to 37 (see additional file 1). From the phenotypic data it was observed that when sown late (on 10 July) the accessions of sorghum generally showed a reduction of growth cycle compared to when sown earlier (on 10 June). The mean DFL50% values of the two sowing dates were significantly different (p < 0.01). For both sowings dates, early-maturing accessions were generally less sensitive to photoperiod (i.e. had lower PRI values), than the late-maturing accessions (which had higher PRI values). The mean plant height of the accessions in the June sowing was significantly (p < 0.001) greater than their mean plant height in the July sowing.

**Table 1:** Days to 50% flowering (DFL50%) and plant height (cm) of sorghum accessions for two sowing dates


	**Sowing 1 (June 10)**			**Sowing 2 (July 10)**			

**Trait**	**Range**	**Mean**	**SE**	**Range**	**Mean**	**SE**	**t**

DFL50%	47-141	99.84	1.05	44-117	79.00	0.76	40.23**
Plant height	132-590	417.74	5.20	112-550	362.86	4.91	8.51***

**, *** Genetic differences among accessions significant at *P *< 0.01 and < 0.001, respectively

### Co-localization of the genes on sorghum genome sequence

Gene sequences of the CGs studied were BLAST-searched against the aligned sorghum genome sequence (Paterson et al. [38]) to identify the physical locations of these genes. BLAST search identified that the *CRY1-b1 *gene has its best possible hit on sorghum chromosome 4 at 53.35 Mb (similar to Sb04g023680), *GI *gene has a unique location on sorghum chromosome 3 at 3.88 Mb (similar to Sb03g003650), *CRY2-2 *gene has a location on sorghum chromosome 6 at 48.11 Mb (similar to Sb06g018510), *LHY *gene is located on sorghum chromosome 4 at 61.55 Mb (similar to Sb04g031590), *HD6 *gene has its best possible hit on sorghum chromosome 2 at 0.98 Mb (similar to Sb02g001110), and *SbD8 *gene has its best possible hit on sorghum chromosome 1at 9.42 Mb (similar to Sb01g010660).

To validate whether these genomic regions have any association with flowering genes like *CRY1b *and *GI*, gene sequences for *CRY1b *and *GI *from different cereals and other model crop species [*CRY1b *sequences from *Oryza sativa *(OsAB073547), *T. aestivum *(TaEF601537), *H. vulgare *(HvDQ201153), *A. thaliana *(AtGQ177026), and *GI *sequences from *O. sativa *(OsAJ133787), *T. aestivum *(TaAF543844), *H. vulgare *(HvAY740524), *A. thaliana *(AtAF105064)] were BLAST searched against the aligned sorghum genome. For each gene, only the best hit could be considered for budget reasons. For the six CGs, an overview of their BLAST scores, E-values with sorghum, and percentage similarity with their respective homologs in *Arabidopsis *and rice obtained by direct nucleotide sequence comparisons is given in Table 2.

**Table 2:** Sorghum candidate genes studied, their predicted and amplified sizes in base pairs,% of gene targeted, BLAST scores and E values with sorghum, their percentage similarities with homologous loci in *Arabidopsis thaliana *(At) and rice (Os) obtained by direct nucleotide sequence comparisons


**Gene**	**Predicted** **size (bp)**	**Fragment** **size (bp)**	**% of gene** **targeted**	**BLAST score** **sorghum**	**E value**	**Similarity** **Arabidopsis %**	**Homologous** **At locus**	**Similarity** **rice %**	**Homologous** **Os locus**

*CRY1-b1*	3954	726	18	1236.4	0	38	NM_116961	50	AB073546
*CRY2-2*	3971	657	17	1160.9	0	57	AY05744	53	AJ298877
*SbD8*	2653	531	27	910.0	0	56	NM_105306	82	AB262980
*Hd6*	6454	807	13	425.1	6.2^e-117^	46	ATHCK2B	52	AB036788
*GI*	8589	960	11	1703.1	0	57	NM_102124	83	AJ133787
*LHY-4*	2110	706	25	1322.0	0	37	NM_001197953	65	NM_001067567

### Candidate gene sequence diversity

Sequences obtained from primers designed in this study (Table 3) were the desired fragments of the targeted gene (see additional file 2). This was confirmed by the high BLAST scores obtained when all fragments were searched against the sorghum genome sequence database (Phytozome) using the BLAST tool. For the CGs, 35% of the total sequenced region (4386 bp) was coding and 65% was non-coding. The number of polymorphic sites was highest for *GI *and lowest for *CRY2-2 *(Table 4). Considering all six genes in this study, the average number of polymorphic sites (*S*) was 12.5, the average nucleotide diversity (π) was 0.005, and Tajima's *D *value was negative for all genes and was highly significant for genes *CRY2-2*, *HD6*, and *GI*.

**Table 3:** Sorghum candidate genes studied, their primer sequences, and primer melting temperatures (T_m_)


**Candidate genes**	**T_m_**	**Forward primer sequences (5'**→**3')** **Reverse primer sequences (5'**→**3')**

*CRY1-b1*	58°C 60°C	ACAACCCAGACTCGCATAG GAGGGATCGAACCGTAGAG
*CRY2-2*	54°C 56°C	ACCTTGTTTCTCCGTTCC CTTCTTGCAGTCTGGCTTT
*LHY-4*	52°C 48°C	CCCTTGACATTGACATAC CATTGATTCCCACTTGA
*HD6*	58°C 64°C	GATTACTGCCATTCACAAGG GAAGCTCAGGWCCCTTGAAGTA
*GI*	58°C 58°C	TCCGCTTCAGCCACCTAC CTGCCAGAGCAATGAGACAA
*SbD8*	60°C 54°C	GACGACAAGGATGAGGAGC CGAGGTGGCGATGAGC

**Table 4:** Sequence diversity of genes *CRY1-b1*, *CRY2-2*, *SbD8*, *HD6*, *GI*, and *LHY-4 *in sorghum


**Gene**	**Fragment size (bp)**	**Predicted gene size (bp)**	**% gene targeted**	***S***	**π**	**Tajima's *D***

*CRY1-b1*	726	3954	18	15	0.002	-1.50
*CRY2-2*	657	3971	17	2	0.001	-2.67**
*SbD8*	528	2653	20	4	0.002	-1.27
*HD6*	804	6454	13	6	0.011	-2.62***
*GI*	960	8589	11	42	0.001	-2.72***
*LHY-4*	706	2110	34	6	0.012	-1.75

For each gene, the total predicted size and the size of amplified fragment, *S *number of polymorphic sites, π the pairwise nucleotide diversity and Tajima's *D *value are reported; **, *** significant at *P *< 0.01 and < 0.001, respectively

### Linkage disequilibrium analyses

**Figure 2:**
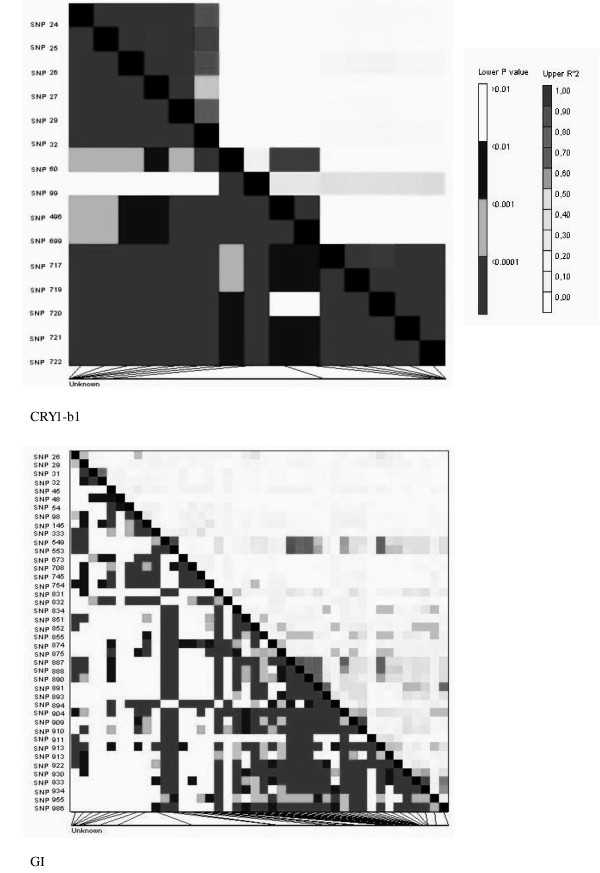
**Strength and extent of linkage disequilibrium for genes *CRY1-b1*, and *GI***. Each point in the linkage disequilibrium matrix represents a comparison between a pair of polymorphic sites, with the r^2 ^values displayed above the diagonal, and *P *values for Fisher's exact test below.

A linkage disequilibrium analysis was performed for six CGs under study. The average r^2 ^values for the CGs were, *CRY1-b1 *= 0.21, *CRY2-2 *= 0.13, *LHY-4 *= 0.074, *HD6 *= 0.31, *GI *= 0.17, and *SbD8 *= 0.024. In the case of the *CRY1-b1 *gene, two strong linkage disequilibrium blocks were detected at the 5' UTR (untranslated region) and at the 3' end of the sequence (coding region). The linkage disequilibrium matrix plots for the CGs studied are shown in Figure 2 and additional file 3.

### Association analyses with candidate genes

Association analyses were performed for all polymorphic sites in all six genes sequenced. Significant *(p *= 0.05) associations were found between PRI and several polymorphic sites within CGs *CRY1-b1 *and *GI *(Table 5 and additional file 4). The SNP722 in *CRY1-b1 *(change of nucleotide base from T to A) and SNP888 in *GI *(change of nucleotide base from T to C) showed effects on PRI of -4.2 and +8 days, respectively. A negative effect on PRI means that the difference in flowering time between the June and July sowing dates was reduced (i.e., photoperiod sensitivity is reduced), whereas a positive effect on PRI indicates that the difference in flowering time was increased (i.e., photoperiod sensitivity is increased).

**Table 5:** Significant (*P *< 0.05) marker-phenotype associations for genes *CRY1-b1* and *GI* in sorghum


**Gene**	**Polymorphism**	**Change of allele state**	**Type of change**	**AE (days)**	**SE (AE)**	***P***

*CRY1-b1*	SNP722	T_A	aa	-4.20	1.51	0.006
*GI*	SNP888	T_C	aa	+7.98	2.90	0.008
*GI*	indel904	0_1	fs	+7.25	2.64	0.008
*GI*	SNP909	C_G	aa	+7.38	2.07	0.001

Note: For change of allele state, 0 denotes the absence and 1 denotes the presence of an indel.

Type of change caused by polymorphism as aa: amino acid substitution or fs: frame-shift mutation, AE: allele effect, SE: standard error of the allelic effect, *P*: *P*-value of allelic effect

## Discussion

### Photoperiod sensitivity

The variability for photoperiod sensitivity observed in this panel of sorghum accessions was very large, ranging from highly insensitive varieties (no change in vegetative period) to highly sensitive (with a 37-day reduction in vegetative cycle induced by the 30-day delay in sowing: from 10 June to 10 July). The accessions that matured earlier in both sowings were mostly the least photoperiod-sensitive ones (having lower PRI values, see additional file 1). Earlier-flowering accessions made the transition from vegetative growth to generative growth before the day-length reached the critical photoperiods required to induce flowering in the later-flowering photoperiod-sensitive accessions. For accessions flowering comparatively late in the June planting, the critical photoperiod significantly reduced flowering time when they were sown under decreasing day-length conditions in July. This was clearly demonstrated by significantly lower mean DFL50% of the July sowing compared to the mean DFL50% of June sowing in these late, photoperiod-sensitive accessions. This reduction in mean DFL50% comes with its consequence, as the mean plant height of the accessions in the July sowing was significantly lower than their mean plant height in the June sowing. Similar observations on reduction in vegetative growth resulting from decreasing day-length conditions were made by Folliard et al. [39] on a guinea sorghum cultivar, where total number of leaves was reduced by half when it was sown at four different sowing dates. The diversity of photoperiod response of our panel of accessions made it an appropriate choice for association analysis for candidate flowering genes.

### Linkage disequilibrium analyses

The linkage disequilibrium measure r^2 ^ranged from 0.024 to 0.21 for the CGs in our study. The mean r^2 ^of 0.18 was comparable to the study on sorghum [40] reporting r^2 ^> 0.1 but lower than the previous study on barley [41] which reported r^2 ^> 0.4. The variability in the range of r^2 ^estimates observed in our study can be due to the fact that linkage disequilibrium estimates vary according to the target genomic region as well as number of polymorphic sites [42,43]. Furthermore, because of limited coverage (small fragment size) of the CGs studied, it seems inappropriate to describe the decay of linkage disequilibrium along each CG. Full length sequencing of the studied and additional important photoperiod CGs will be necessary to describe patterns of linkage disequilibrium in the sorghum flowering time gene network.

### Population structure and association analysis

The CGs chosen for this study were selected on the basis of comparative genomic studies on photoperiodic flowering time genes in *A. thaliana *and rice [44-46]. From these studies it was evident that the respective genes have a high degree of similarity in structure and function between the latter two species.

Using software STRUCTURE to infer population structure, and employing Evanno's method to estimate the number of subgroups, our sorghum germplasm panel could be divided into two subgroups [47]. Race as well as the geographical origin of the accessions, played a role in the population structure of these sorghum accessions. We used a mixed-model method for association analysis that takes into account population structure as well as kinship information. This model has proven to yield better results in association studies compared to models ignoring these factors [48,49]. The marker-phenotype association analysis was carried out using values of PRI for each accession. The field experiments were conducted in one year (2007) and at one location. Significant genotype × year interactions for measures of photoperiod-sensitive flowering response might occur in multi-location trials over years; however, the sorghum accessions of our study have been observed previously for their photoperiodic behavior over years and therefore some background information on their photoperiod response was known.

Out of the six genes analyzed, we detected in fragments of genes *CRY1-b1 *and *GI *several polymorphic sites that were significantly (*p *< 0.05) associated with PRI variation in our sorghum panel. The first two candidate genes considered in our study were *CRY1-b1 *and *CRY2-2*. In plants, cryptochromes and phototropins [50] are the two types of blue light/UV-A receptors important for plant photomorphogenesis. In *A. thaliana*, *CRY1 *mainly functions in de-etiolation [51], while *CRY2 *plays a role in the regulation of photoperiodic flowering [52]. Hirose et al. [53] showed that over-expression of *OsCRY1 *in rice resulted in enhanced responsiveness to blue light, suggesting that *OsCRY1 *is a regulator of photomorphogenesis, similar to *AtCRY1*. Like *AtCRY2*, *OsCRY2 *is also involved in the promotion of flowering time in rice [53]. But it was also shown that sub-cellular localization of *AtCRY2 *does not change in response to blue light [54]. In our analysis, we did not find any significant associations of the *CRY2-2 *gene with PRI but several polymorphisms in the *CRY1-b1 *gene were significantly associated with PRI, where the most important polymorphisms showed an effect on PRI value of up to -4.2 days (Table 5).

The *CRY1 *gene sequence in sorghum (*SbCRY1*) has three important domains namely i) DNA photolyase - binding a light harvesting cofactor [54], ii) FAD (flavin adenine dinucleotide) binding domain of the DNA photolyase - involved in energy harness of blue light [55], and iii) blue/ultraviolet-sensing protein C terminal - this domain is found in association with two previous domains in eukaryotes [56]. Our BLAST results showed that the *CRY1-b1 *gene fragment that we analyzed was located between the first domain (DNA photolyase) and the beginning of second domain (FAD domain) of the *SbCRY1 *gene. The SNP at position 722 in *CRY1-b1 *was therefore located in the domain of the DNA photolyase located at the N-terminal domain of *SbCRY1*. In *A. thaliana*, it was shown that the N-terminal domain of the *CRY1 *gene was essential for blue light reception [57]. This domain catalyzes the repair of photo-damage to the light-harvesting apparatus resulting from ultraviolet irradiation. Photolyases and cryptochromes are related flavoproteins that bind FAD. Photolyases harness the energy of blue light and cryptochromes (*CRY1 *and *CRY2*) mediate blue light-induced gene expression [58]. Therefore the effect of the SNP at position 722 at the N-terminal in our *SbCRY1-b1 *sequence suggested that the change in nucleotide base from T to A (Table 5) might play an important role in blue light reception in sorghum. This observation can be supported by the fact that in wheat the N-terminal domain of *TaCRY1 *contains a sequence signal important for its nuclear export. Therefore, a detailed analysis of *SbCRY1 *comparing its N-terminal domain with its C-terminal domain might reveal their exact roles in photomorphogenesis.

In addition to *CRY1-b1*, we found several polymorphic sites in the sorghum *GI *gene homolog to be significantly associated with PRI, with the largest effect on PRI of about 8 days (Table 5). Hayama et al. ([59,60]) reported that in rice, rather than promoting flowering, *OsGI *expression results in the suppression of flowering under LD. It has been proposed that genetic mechanisms of photoperiodic control in rice are similar to those in *A. thaliana*, but vary in downstream signaling of *GI*, at the regulation of *FT*. In LD conditions, *CO *promotes flowering through *FT *activation in *A. thaliana *and conversely represses *FT *and flowering in rice, which is a SD plant [60]. Similar to the observations in rice [59,60], the positive allele effect on PRI observed in this study (Table 5) indicates that *SbGI *enhances photoperiodic response to SD conditions in sorghum, i.e., *SbGI *shortens the time to sorghum flowering in the later July sowing which is more exposed to SD conditions, while in the June sowing (initially more exposed to LD conditions), *SbGI *delays sorghum flowering. Therefore, detailed investigation by comparison of accessions grown under SD and LD conditions would be necessary to determine the exact mode of action of the *GI *gene homolog in sorghum. Tajima's *D *values for the gene fragments sequenced in our study were negative (Table 4) with three genes (*CRY2-2*, *HD6*, and *GI*) having significantly negative values. Possible reasons for negative Tajima's *D *values (obvious through large numbers of low frequency variants) may be, firstly, that the sorghum accessions of our study originated from different geographical regions and had little common history. Secondly, it has been suggested that population structure existing among the ancestral populations as a result of multiple domestications and introgressions from wild relatives could give rise to negative Tajima's *D *values (see [61]). Thirdly, the negative Tajima's *D *values might indicate that the gene fragments used in our study may have been subjected to adaptive selection as variation in flowering time may confer adaptive advantages in sorghum (see [62]).

## Conclusions

When looking at the flowering time gene network as a whole, purifying selection is found in both coding and non-coding DNA regions [63,64]. The sorghum sequence dataset of our study is in agreement with this observation. Certainly, conclusions can be drawn from Tajima's *D *estimates found in our study about natural selection affecting the studied sorghum genomic regions. However, in our study the number of genes as well as the size of the each CG fragment studied was small for effectively capturing the signature of selection on photoperiodic flowering time genes. It will be necessary to characterize the entire flowering time gene network in sorghum to know how selection has shaped the photoperiod pathway of flowering time and thus helped sorghum to adapt to climatic zones with different day-length conditions.

To utilize the SNPs identified to be significantly associated with PRI in our study, molecular markers could be designed based on coinciding endonuclease restriction sites which in turn could be used to create cleaved amplified polymorphic sequence (CAPS) markers [65]. Furthermore, functional markers could be created directly from the significant SNPs. These markers can thus serve as powerful tools for MAS in sorghum to identify accessions or segregants having specific sensitivities to photoperiod.

## Methods

### Plant material and phenotypic evaluation

Our study was based on 219 inbred accessions of sorghum mainly of the Guinea race (additional file 1), which were grown at the International Crops Research Institute for the Semi-Arid Tropics (ICRISAT) research station at Samanko, Mali in 2007 [47]. The entire panel of accessions was shown on two dates (10^th ^June and 10^th ^of July, respectively) flanking the summer solstice, with two replications each. DFL50% was recorded for each plot as the date when 50% of the plants had at least half of the panicle in anthesis (Table 1). Plant heights of the accessions were measured for both sowings dates. The photoperiod response index (PRI) for each accession was considered as the number of days difference in mean DFL50% between the 1^st ^sowing and 2^nd ^sowing and was calculated using following formula:

$$\text{PRI}=\text{DFL50}{\text{\% }}_{1}-\text{DFL50}{\text{\% }}_{2}$$

where DFL50%_1 _and DFL50%_2 _are the mean days to 50% flowering observed for the first sowing date and second sowing date, respectively, with all DFL50% values expressed in days after sowing. Values close to zero indicate non-photoperiod-sensitive flowering (stable vegetative period); values close to 30 (the difference between the first and second sowing dates) or even higher indicate high sensitivity to photoperiod (sharp shortening of the vegetative period with the later sowing date, and its associated shorter photoperiods).

### Candidate gene sequencing

Primers were designed for the desired regions in CGs *CRY1 *(fragment designation: *CRY1-b1*), *CRY2 *(fragment designation: *CRY2-2*)*, LHY *(fragment designation: *LHY-4*), *GI*, *HD6*, and *SbD8 *[66] based on sequences published in public databases (NCBI and Phytozome) using Primer Premier Software (Premier Biosoft International, Palo Alto, CA, USA). The amplified regions of the CGs were selected on the basis of best possible primer combinations (with minimum secondary structures such as primer dimers and hairpins) and with optimum product size. Primer sequences and their respective melting temperatures are given in Table 3. Besides these six CGs, primers were also designed for partial amplification of other potentially important photoperiod genes such as *Phytochrome A, B *and *C*. Because of the lack of polymorphisms within the amplified fragments, these genes were not considered for further analysis. PCR reactions were performed and PCR products were sequenced by QIAGEN (Hilden, Germany). The gene fragments were sequenced by an easy read sequencing service using ABI BigDye Terminator 3.1 chemistry on a capillary automatic sequencing device (3730xl ABI 96; Applied Biosystems/Applera, Darmstadt, Germany). In our study, the best sequencing results by the easy read sequencing service were obtained for the fragment sizes ± 800 base pairs. Therefore, the CGs included in our study were partially amplified to fit in this range. The sequences obtained were manually checked for allele calling errors and edited manually by using software Chromas [67]. The gained nucleotide sequence data were deposited in the NCBI GenBank under the following accession numbers: *CRYPTOCHROME *1 (*CRY1*; Sb04g023680; [NCBI GenBank accession number: JQ350839]), *CRYPTOCHROME *2 (*CRY2*; Sb06g018510; [GenBank: JQ350840]), *LATE ELONGATED HYPOCOTYL *(*LHY*; Sb04g031590; [GenBank: JQ350844]), *GIGANTEA *(*GI*; Sb03g003650;[GenBank: JQ350842]), *HEADING DATE *6 (*HD6; *Sb02g001110; [GenBank: JQ350843]), and *Dwarf8 *(*SbD8*; Sb01g010660; [GenBank: JQ350841]).

For further analysis of the sequenced genes, multiple alignments of the sequences were done by using software program ClustalW2 [68]. For CGs, the number of polymorphic sites (S), pairwise nucleotide diversity (π), and Tajima's *D *[69] values, were computed using DnaSP [70]. For the CGs, the linkage disequilibrium matrix plots (Figure 2 and additional file 2) of r^2 ^(squared correlation coefficient) values against the pair-wise physical distance between polymorphic sites were obtained with software TASSEL [71].

### Association analyses

The population structure of the diversity panel was previously determined by the software STRUCTURE (Bhosale et al. [47]) and its Q matrix employed herein for association analysis. This was done by setting the number of subgroups from 1 to 20 with five runs, allowing for the admixture, correlated allele frequencies and no recombination information. For each run of STRUCTURE, the burn-in time as well as the iteration number for the Markov chain Monte Carlo algorithm was set to 100,000.

The QK method described by Yu et al. [48] was used for detection of marker-phenotype associations:

$$M_{ip}=\mu +\sum Q_{iu}v_{u}+a_{p}+\bar{g_{i}}+e_{ip}$$

where *M_ip _*is the adjusted entry mean of the *i*^th ^sorghum inbred carrying the *p*^th ^allele, *μ *is an intercept term, *v_u _*the effect of the *u*^th ^column of the population structure matrix *Q*, *a_p _*the effect of allele *p*, *g_i _*the genetic effect of the *i*^th ^sorghum inbred in addition to *a_p_*, and *e_ip _*is the residual [49]. The variances of the random effects *g *= {*g*_1_, ... *g*_219_} and *e *= *{e*_1, 1_,..., *e*_209, 2_*} *were assumed to be $\text{var}\left(g\right)=2\text{K}\sigma_{\bar{g}}^{\text{2}}$ and $\text{var}\left(e\right)=\text{I}\sigma_{r}^{\text{2}}$, where K was a 219 × 219 matrix of kinship coefficients that define the degree of genetic covariance between all pairs of entries and was calculated using SPAGeDi [72]. Estimates of$\sigma_{\bar{g}}^{\text{2}}$, the genetic variance and$\sigma_{r}^{\text{2}}$, the residual variance, were obtained by REML. All mixed-model calculations were performed with ASReml release 2.0 [73].

## List of abbreviations

PRI: Photoperiod response index; SNPs: Single nucleotide polymorphisms; *CRY1-b1*: *CRYPTOCHROME 1*; *GI*: *GIGANTEA*; WCA: Western and Central Africa; SD: Short-day; LD: Long-day; *CCA1*: *CIRCADIAN CLOCK ASSOCIATED1*; *LHY*: *LATE ELONGATED HYPOCOTYL*; *TOC1*: *TIMING OF CAB EXPRESSION1*; *CO*: *CONSTANS*; *FT*: *FLOWERING LOCUS T*; CGs: Candidate genes; *HD6*: *HEADING DATE *6; *SbD8*: *Dwarf8*; DFL50%: Days to 50% flowering; UTR: Untranslated region; FAD: Flavin adenine dinucleotide; CAPS: Cleaved amplified polymorphic sequence; ICRISAT: International Crops Research Institute for the Semi-Arid Tropics.

## Competing interests

The authors of the manuscript entitled 'Association analysis of photoperiodic flowering time genes in west and central African sorghum [*sorghum bicolor *(L.) Moench]', declare that they have no competing interests.

## Authors' contributions

HKP, BIGH designed and HKP and AEM supervised the research; FR and EW conducted the field trials, SUB conducted the molecular work and BS and SUB analyzed the data. PR, CTH, AP and HC identified locations of CGs in the sorghum genome and extensively revised the manuscript. SUB, HKP, BS, BIGH, PR, CTH and FR wrote the manuscript. All authors except HKP read and approved the final manuscript.

## Supplementary Material

Additional file 1 **List of sorghum accessions, days to 50% flowering (DFL50%) for June and July sowings, their photoperiod response indices (PRIs), races and countries of origin**. Click here for file

Additional file 2 **Amplified fragments of sorghum candidate genes blasted against sorghum genome database**. Click here for file

Additional file 3 **Strength and extent of linkage disequilibrium for genes *CRY2-2*, *SbD8, HD6*, and *LHY4***. Each point in the linkage disequilibrium matrix represents a comparison between a pair of polymorphic sites, with the r^2 ^values displayed above the diagonal, and *P *values for Fisher's exact test below. Click here for file

Additional file 4 **Association of genes *CRY1-b1*, *CRY2-2*, *SbD8, GI*, *HD6*, and *LHY4 *with photoperiod response index (PRI) in sorghum**. Click here for file
