# Supplementary material for: Association analysis of photoperiodic flowering time genes in west and central African sorghum [Sorghum bicolor (L.) Moench]
Source: BMC Plant Biol. 2012 Mar 7;12:32. doi: 10.1186/1471-2229-12-32 (PMC3364917; doi:10.1186/1471-2229-12-32)
Supplement: Additional file 2 — Amplified fragments of sorghum candidate genes blasted against sorghum genome database. [file 1471-2229-12-32-S2.DOC]

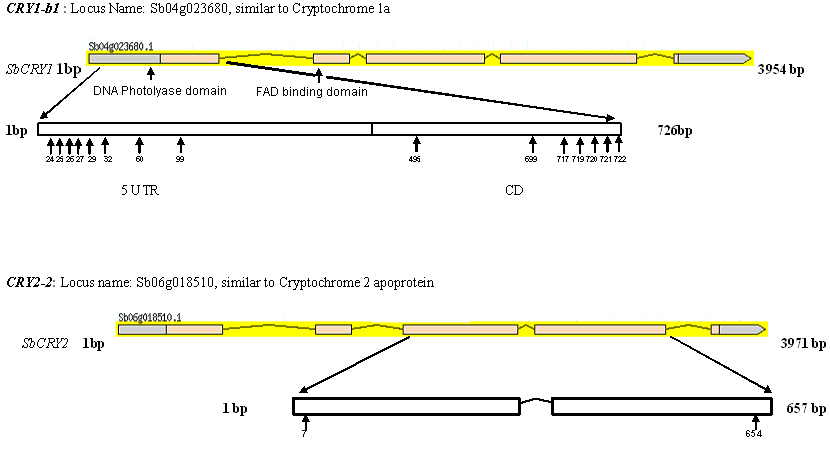


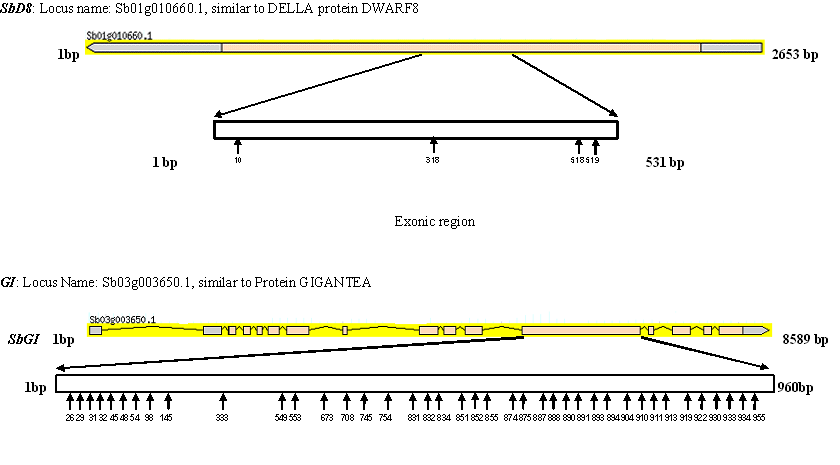


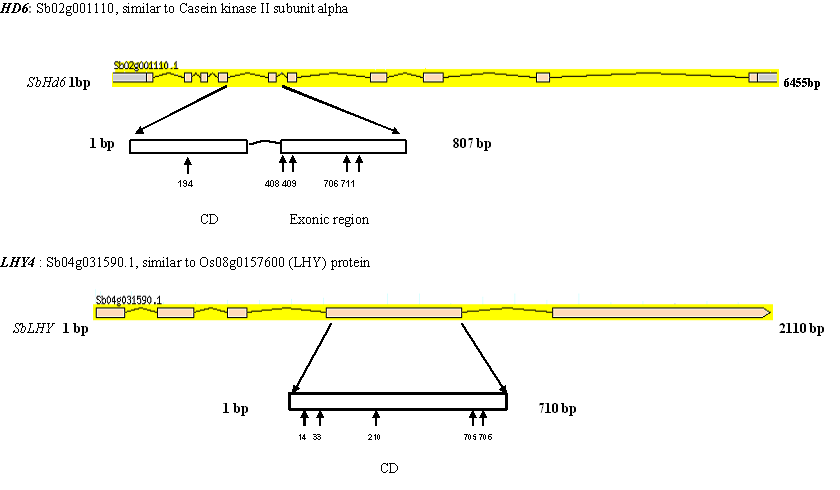


Additional file 2 Amplified fragments of sorghum candidate genes blasted against sorghum genome database (http://www.phytozome.net/search.php?show=blast&method=Org_Sbicolor). SNPs are denoted by arrows on the top of numbers denoting the position of each SNP and CD refers to coding sequences. Colored fragments are genomic regions of sorghum genome including regions showing similarity to the amplified fragments in the study.
