# Supplementary material for: Association analysis of photoperiodic flowering time genes in west and central African sorghum [Sorghum bicolor (L.) Moench]
Source: BMC Plant Biol. 2012 Mar 7;12:32. doi: 10.1186/1471-2229-12-32 (PMC3364917; doi:10.1186/1471-2229-12-32)
Supplement: Additional file 3 — Strength and extent of linkage disequilibrium for genes CRY2-2, SbD8, HD6, and LHY4. Each point in the linkage disequilibrium matrix represents a comparison between a pair of polymorphic sites, with the r2 values displayed above the diagonal, and P values for Fisher's exact test below. [file 1471-2229-12-32-S3.DOC]

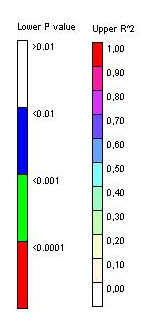

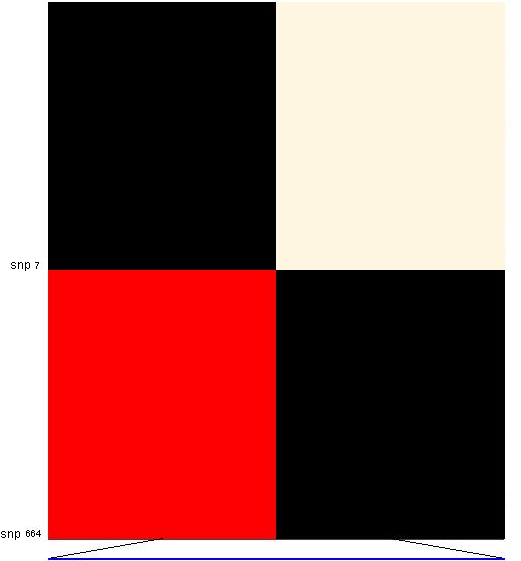


*CRY2-2*

*
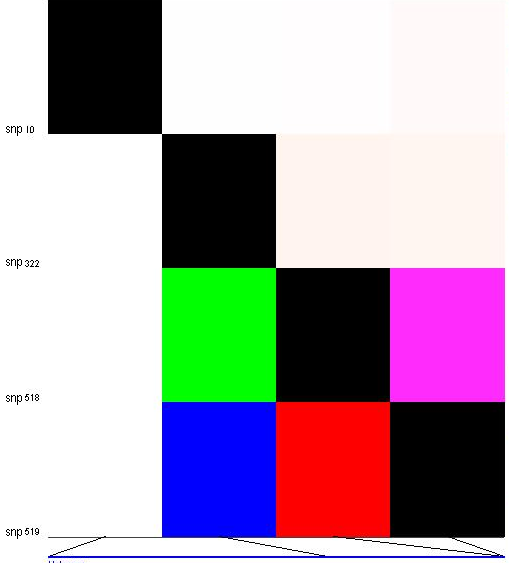
*

*SbD8*

*
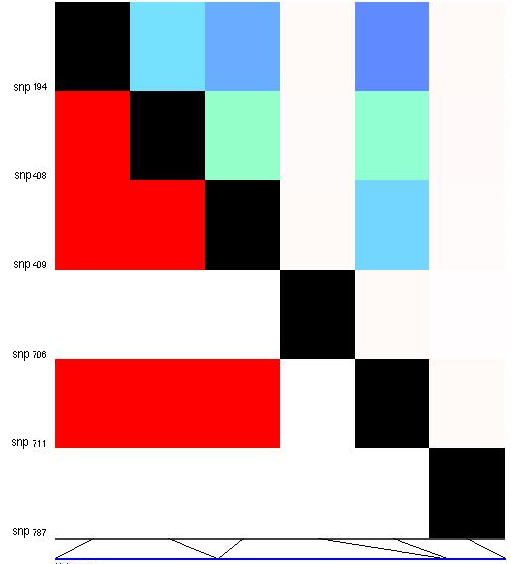
*

*HD6*


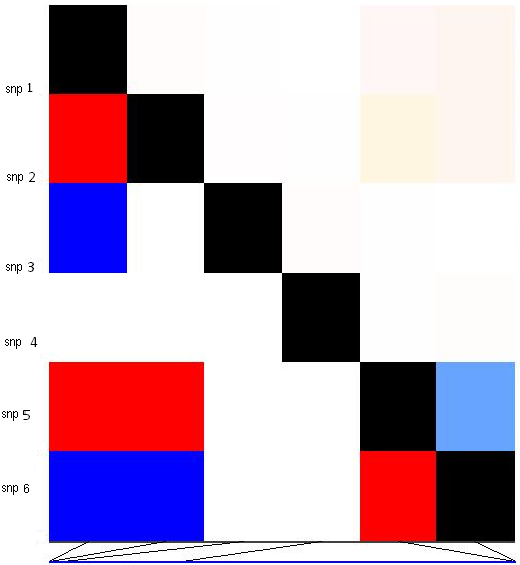


*LHY-4*

Additional file 3 -Strength and extent of linkage disequilibrium for genes *CRY2-2*, *SbD8,* *HD6*, and *LHY4*. Each point in the linkage disequilibrium matrix represents a comparison between a pair of polymorphic sites, with the r^2^ values displayed above the diagonal, and *P* values for Fisher’s exact test below
