# Supplementary material for: Association analysis of photoperiodic flowering time genes in west and central African sorghum [Sorghum bicolor (L.) Moench]
Source: BMC Plant Biol. 2012 Mar 7;12:32. doi: 10.1186/1471-2229-12-32 (PMC3364917; doi:10.1186/1471-2229-12-32)
Supplement: Additional file 4 — Association of genes CRY1-b1, CRY2-2, SbD8, GI, HD6, and LHY4 with photoperiod response index (PRI) in sorghum. [file 1471-2229-12-32-S4.DOC]

Additional file 4 Association of genes *CRY1-b1*, *CRY2-2*, *SbD8,* *GI*, *HD6*, and *LHY4*

with photoperiod response index (PRI) in sorghum.

| Gene | Polymorphic  site position | Change of  Allele state | Allele effect | SE | *P* value |
| --- | --- | --- | --- | --- | --- |
| *CRY1-b1* | SNP24 | T_C | -1.21 | 1.17 | 0.300 |
| *CRY1-b1* | SNP25 | G_C | -1.07 | 1.11 | 0.334 |
| *CRY1-b1* | SNP26 | A_G | -2.08 | 1.28 | 0.105 |
| *CRY1-b1* | SNP27 | T_A | -1.05 | 1.12 | 0.377 |
| *CRY1-b1* | SNP29 | T_C | -0.73 | 1.08 | 0.497 |
| *CRY1-b1* | Indel32 | 0_1 | -0.26 | 1.06 | 0.801 |
| *CRY1-b1* | Indel60 | 1_0 | -0.45 | 1.50 | 0.763 |
| *CRY1-b1* | Indel99 | 1_0 | -2.67 | 1.10 | 0.016 |
| *CRY1-b1* | SNP496 | G_A | -1.97 | 1.55 | 0.207 |
| *CRY1-b1* | SNP699 | G_C | -1.96 | 1.55 | 0.207 |
| *CRY1-b1* | Indel717 | 1_0 | -3.51 | 1.46 | 0.017 |
| *CRY1-b1* | SNP719 | T_C | -3.37 | 1.46 | 0.021 |
| *CRY1-b1* | SNP720 | T_C | -3.40 | 1.46 | 0.020 |
| *CRY1-b1* | SNP721 | T_C | -3.77 | 1.53 | 0.014 |
| *CRY1-b1* | SNP722 | T_A | -4.20 | 1.51 | 0.006 |
| *CRY2-2* | SNP7 | C_A | -0.57 | 1.04 | 0.589 |
| *CRY2-2* | indel664 | 1_0 | 0.09 | 1.12 | 0.937 |
| *SbD8* | SNP10 | T_C | +0.57 | 1.81 | 0.753 |
| *SbD8* | SNP322 | T_C | +2.49 | 1.50 | 0.098 |
| *SbD8* | SNP518 | A_C | +0.76 | 1.40 | 0.590 |
| *SbD8* | SNP519 | C_A | +0.87 | 1.29 | 0.502 |
| *GI* | indel26 | 0_1 | -0.55 | 1.82 | 0.761 |
| *GI* | indel29 | 1_0 | -1.53 | 1.79 | 0.396 |
| *GI* | SNP31 | T_G | -2.87 | 2.84 | 0.316 |
| *GI* | SNP32 | A_T | -3.58 | 2.60 | 0.174 |
| *GI* | indel45 | 1_0 | +0.30 | 1.96 | 0.878 |
| *GI* | SNP48 | A_G | +0.52 | 4.88 | 0.916 |
| *GI* | SNP54 | T_G | -2.50 | 3.48 | 0.476 |
| *GI* | SNP145 | G_A | -2.50 | 1.78 | 0.165 |
| *GI* | SNP333 | T_A | +2.07 | 3.18 | 0.518 |
| *GI* | indel549 | 0_1 | +3.35 | 2.62 | 0.206 |
| *GI* | indel553 | 0_1 | +3.35 | 2.62 | 0.206 |
| *GI* | SNP673 | T_G | -1.20 | 2.55 | 0.638 |
| *GI* | SNP708 | G_T | +1.48 | 2.70 | 0.586 |

| Gene | Polymorphic  site position | Change of  Allele state | Allele effect | SE | *P* value |
| --- | --- | --- | --- | --- | --- |
| *GI* | SNP745 | G_C | +1.72 | 2.17 | 0.430 |
| *GI* | SNP754 | T_G | -0.22 | 1.72 | 0.901 |
| *GI* | indel831 | 1_0 | -1.29 | 1.70 | 0.453 |
| *GI* | SNP832 | G_C | +1.70 | 2.49 | 0.496 |
| *GI* | indel834 | 1_0 | -0.71 | 1.75 | 0.686 |
| *GI* | indel852 | 1_0 | -2.73 | 2.15 | 0.209 |
| *GI* | indel855 | 0_1 | +0.15 | 1.89 | 0.939 |
| *GI* | indel874 | 1_0 | +1.06 | 1.80 | 0.558 |
| *GI* | indel875 | 1_0 | +1.06 | 1.80 | 0.558 |
| *GI* | indel887 | 0_1 | +6.57 | 3.12 | 0.039 |
| *GI* | SNP888 | T_C | +7.98 | 2.90 | 0.008 |
| *GI* | SNP890 | T_C | +0.77 | 2.03 | 0.704 |
| *GI* | SNP891 | C_A | -1.19 | 2.09 | 0.574 |
| *GI* | SNP893 | A_C | -1.19 | 1.87 | 0.525 |
| *GI* | indel904 | 0_1 | +7.25 | 2.64 | 0.008 |
| *GI* | SNP909 | C_G | +7.38 | 2.07 | 0.001 |
| *GI* | SNP910 | G_C | +4.84 | 2.00 | 0.02 |
| *GI* | SNP913 | G_C | +2.62 | 2.64 | 0.326 |
| *GI* | SNP922 | T_C | +3.14 | 1.64 | 0.059 |
| *GI* | SNP933 | T_C | +1.42 | 1.79 | 0.431 |
| *GI* | SNP934 | G_A | -0.13 | 1.83 | 0.945 |
| *GI* | SNP955 | T_G | -1.88 | 2.47 | 0.450 |
| *HD6* | SNP408 | T_G | +2.12 | 1.24 | 0.090 |
| *HD6* | SNP409 | G_T | +2.58 | 1.40 | 0.066 |
| *HD6* | SNP706 | C_T | -0.34 | 1.02 | 0.737 |
| *HD6* | SNP711 | A_T | -0.63 | 1.33 | 0.633 |
| *HD6* | indel780 | 0_1 | +1.62 | 1.29 | 0.209 |
| *LHY4* | indel14 | 0_1 | -0.93 | 1.41 | 0.513 |
| *LHY4* | indel33 | 1_0 | +2.15 | 1.11 | 0.053 |
| *LHY4* | SNP210 | T_C | +0.26 | 1.57 | 0.868 |
| *LHY4* | SNP705 | T_G | -0.08 | 1.00 | 0.939 |
| *LHY4* | SNP706 | G_A | +0.07 | 1.25 | 0.954 |

Note: For change of allele state 0 denotes the absence and 1 denotes the presence of an indel;

Type of change caused by polymorphism as aa: amino acid substitution or fs: frame-shift mutation; AE: allele effect; SE: standard error of the allelic effect; *P*: *P*-value of allelic effect.
